# Electrophysiological correlates of prediction formation in anticipation of reward‐ and punishment‐related feedback signals

**DOI:** 10.1111/psyp.13379

**Published:** 2019-04-26

**Authors:** Miles Wischnewski, Dennis J.L.G. Schutter

**Affiliations:** ^1^ Donders Institute for Brain, Cognition and Behaviour Radboud University Nijmegen Nijmegen The Netherlands

**Keywords:** ERP, feedback processing, medial frontal negativity, performance monitoring, P2, P3

## Abstract

Feedback processing during decision making involves comparing anticipated and actual outcome. Although effects on ERPs of valence, magnitude, expectancy, and context during feedback processing have been extensively investigated, the electrophysiological processes underlying prediction formation in anticipation of feedback signals have received little attention. The aim of the present study was to explore these processes of prediction formation and their influence on subsequent feedback signals. Twenty healthy, right‐handed volunteers performed a forced‐choice task in which they had to indicate which of two presented objects was more expensive. After the volunteer's choice, an expert cue, which was accurate in 80% of trials, was presented to manipulate prediction formation about future reward and punishment. ERPs were recorded during presentation of the expert cue and during feedback. Results revealed that prediction formation of future rewards and punishments is accompanied by differences in the P2 component and a subsequent delay period. During feedback processing, the prediction‐related P2 was associated with the processing of valence reflected in the feedback‐related P2. Furthermore, the prediction‐related difference in the delay period was associated with error processing in feedback‐related medial frontal negativity. These findings suggest that prediction signals prior to feedback contain information about whether a prediction is correct or wrong (expectancy) and if the outcome will be a reward or punishment (valence).

## Abstract 2

During feedback processing, a prediction of future outcome is affected by contextual information. Here, we show that electrocortical signals related to future outcome following contextual information inversely predict reward signals during feedback processing. Additionally, signal differences after presentation of contextual information relate to future signal differences reflecting prediction error during feedback processing.

## 1 INTRODUCTION

The predictive coding account of decision making postulates that decisions are followed by a prediction about the subsequent outcome and that this prediction is evaluated during feedback processing (Alexander & Brown, 2018; Friston, 2005; Friston & Kiebel, 2009; Rushworth, Mars, & Summerfield, 2009; Van Pelt et al., 2016). A mismatch between prediction and actual outcome, a prediction error, is thought to prompt minimization of the observed error (Holroyd, Nieuwenhuis, Yeung, & Cohen, 2003). Context is important as it guides the anticipated outcome prediction and thus the subsequent prediction error (Hajcak, Holroyd, Moser, & Simons, 2005; Holroyd, Larsen, & Cohen, 2004; Hajcak, Moser, Holroyd, & Simons, 2007; Kahneman & Tversky, 1979; Tversky & Kahneman, 1992; Wischnewski & Schutter, 2018). For example, an expert cue can strongly shape participants' expectations and guide decision making (Meshi, Biele, Korn, & Heekeren, 2012; Wischnewski, Bekkering, & Schutter, 2018). On the one hand, if a contextual cue hints toward a positive outcome, a reward will not cause a prediction error. On the other hand, in a contextual environment that points toward a negative outcome, a reward will elicit a prediction error.

ERP studies have demonstrated that prediction‐outcome mismatch detection and error minimization during the processing of feedback are associated with distinct exogenous and endogenous electrophysiological components (Fischer & Ullsperger, 2013; Holroyd et al., 2003). First, the P1 and N1, which peak around 100 ms after feedback presentation over the occipito‐parietal cortex, are exogenous visual components. Trautmann‐Lengsfeld and Herrmann (2013) provided evidence that even such early visual attention components can be influenced by contextual advice cues. They found decreased P1 amplitudes when participants followed incorrect advice compared to correct advice, which suggests that bottom‐up attention can be biased by the prediction of rewards and punishments (Trautmann‐Lengsfeld & Herrmann, 2013). Second, the P2 is a brain potential that peaks over fronto‐central electrodes approximately 200 ms after feedback presentation and is linked to early attentional processes that discriminate between reward and punishment signals. In a previous study, we showed that the P2 is modulated by context. We found that an outcome that is in relative terms perceived as a punishment elicits increased P2 amplitudes, even though in absolute terms this outcome can be interpreted as a reward (Wischnewski & Schutter, 2018). Third, the medial frontal negativity (MFN; also referred to as feedback‐related negativity) peaks between 200 and 300 ms after feedback onset over fronto‐central electrodes and shows a larger amplitude when the predicted outcome does not match the actual outcome (Holroyd et al., 2003). Results from a number of studies indicate that the MFN primarily reflects the detection of a discrepancy between anticipated reward and reward omission. According to this view, the MFN is proposed to reflect a process in which worse‐than‐expected outcomes are distinguished from expected and better‐than‐expected outcomes (Bellebaum, Polezzi, & Daum, 2010; Hajcak et al., 2005, 2007; Holroyd et al., 2003). Yet, other studies indicate that rewards may yield increased MFN amplitudes compared to punishments if this is an uncommon or surprising result. Therefore, these findings suggested that the MFN indexes an action‐outcome predictor that encodes unexpected results independent of valence (Alexander & Brown, 2011, 2018; Jessup, Busemeyer, & Brown, 2010; Talmi, Atkinson, & El‐Deredy, 2013). Finally, a parietal positive peak around 300–500 ms (P3) is thought to reflect top‐down attentional allocation toward perceived outcome mismatches. Indeed, the P3 has been linked to the fronto‐parietal attention network as observed in fMRI studies (Bengson, Kelley, & Mangun, 2015; Pfabigan et al., 2014). Fischer and Ullsperger (2013) showed that the P3 is involved in the updating of context and future predictions based on feedback, by means of error minimization, constituting a learning effect.

Whereas the electrophysiological processes during feedback processing have been well studied, less is known about the electrocortical signals of prediction formation and how these predictions affect subsequent feedback processing. A slow wave component that is frequently described in the literature during anticipation of feedback is the stimulus preceding negativity (SPN). The SPN is thought to reflect the anticipation about informational (correct or incorrect response) and motivational (win or loss) aspects of future feedback (Brunia, van Boxtel, & Böcker, 2012; Van Boxtel & Böcker, 2004). Yet, to date, it is not known if this component relates to prediction and errors in prediction of upcoming feedback. Furthermore, Stefanics and colleagues (2010) investigated the processing of predictions in the sensory domain. In their experiment, the occurrence of a target auditory stimulus could be predicted by the frequency of a previous auditory cue. They observed slow potentials in the delta (0.5–3 Hz) frequency range prior to the onset the target stimulus, which showed higher amplitudes after more predictable cues. It has been proposed that a prediction signal contains information about expected outcome (i.e., whether the outcome will match the prediction) as well as aspects of valence attached to this future outcome (i.e., whether the outcome will be positive or negative; Summerfield & Egner, 2009; Summerfield et al., 2006). To date, it is unclear whether these results from the visual domain can be extrapolated to the cognitive domain of decision making.

The aim of the present study was to explore the electrophysiological correlates of prediction formation and the effects on the processing of subsequent feedback signals. Specifically, three main hypotheses were formulated: (a) higher P2 and P3 amplitudes were expected to reward‐ as compared to punishment‐related feedback; (b) this anticipated signal difference, during feedback processing, would be positively correlated to signal differences between expectation of reward and punishment, during expert cue processing; and (c) unexpected outcomes would produce a prediction error as reflected by a larger MFN amplitude. In addition to the main hypotheses, we explored the signal differences and oscillatory power within the delay period, and it was expected that these would positively correlate to prediction error or valence processing during feedback processing. Since no SPN was observed, it is not further considered in the present results.

## 2 METHOD

### 2.1 Participants

Twenty healthy volunteers (14 female, mean age ± *SD*: 22.7 ± 3.8) participated in the present study. All participants were right‐handed (mean ± *SD*, 44.1 ± 3.7) as determined by the Edinburgh Handedness Inventory (Oldfield, 1971) and had normal or corrected‐to‐normal vision and no history of neurological or psychiatric disorders. The study protocol was approved by the local ethical committee of the Donders Centre for Cognition in Nijmegen and carried out in accordance with the standards set by the Declaration of Helsinki (Fortaleza Amendments).

### 2.2 Decision‐making task

**Figure 1:**
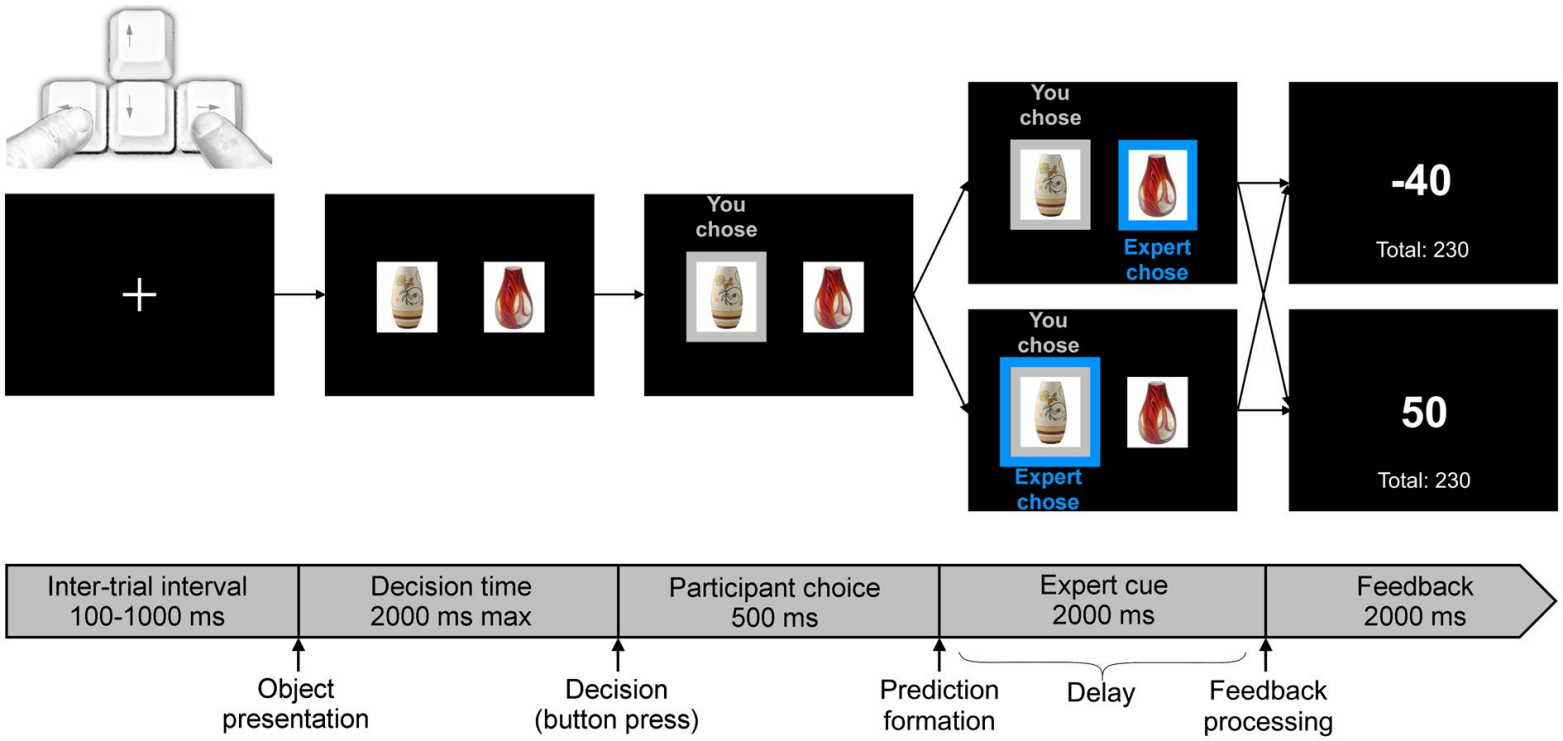
Example of a single trial of the decision‐making task. Two vases were shown, and participants had to indicate which of the two was the more expensive using left or right index finger. A response was followed by a highly valid expert cue (80% correct). Finally, reward (+50 points) and punishment (−40 points) feedback was shown

In this task, participants were shown two horizontally presented vases (resolution 350 × 250, presented 5 cm left and right of the center, visual angle 3.5°) on a screen (22‐in., 30 × 48 cm, resolution: 1,680 × 1,050) and had to indicate which was the more expensive by pressing the left or right response button with their index fingers (maximum response time: 2,000 ms). Vase pictures had a white background and were presented on a black screen. The stimuli were gathered from a database that was used in previous studies of our group (Wischnewski, Bekkering, & Schutter, 2018; Wischnewski & Schutter, 2017). After a choice was made, the selected vase was surrounded by a gray square with the text, “You chose” above it. After 500 ms, an additional square with blue color appeared with the text, “Expert chose” above it. This expert cue was programmed to be correct in 80% of the trials, and this information was shared with participants before the start of the task. Participants were informed that they could use the expert choice to make a prediction for subsequent reward (+50 points) or punishment (−40 points). Different vase stimuli were used in all trials to avoid learning effects and to ensure attentional focus of the participants on the expert cue. Having the same answer as the expert would in 80% of trials result in a reward, whereas having chosen the other option would in the majority of cases result in a punishment. The task therefore yielded four conditions (Figure 1): (a) congruent reward: Expert agreed with participant's choice, meaning that the subject would expect a reward, followed by indeed receiving a reward. The outcome is therefore congruent with the prediction; (b) congruent punishment: Expert disagreed with participant's choice, meaning that the subject would expect a punishment, followed by indeed receiving a punishment. The outcome is therefore congruent with the prediction; (c) incongruent reward: Expert disagreed with participant's choice, meaning that the subject would expect a punishment, followed by receiving a reward. The outcome is therefore incongruent with the prediction; (c) incongruent punishment: Expert agreed with participant's choice, meaning that the subject would expect a reward, followed by receiving a punishment. The outcome is therefore incongruent with the prediction. In a previous study, using a similar task with the same vase stimuli, we found that participants do not score above chance level and strongly rely on external cues (Wischnewski, Bekkering, & Schutter, 2018). Therefore, in this study and unknown to the participants, outcomes were fixed with 50% reward trials and 50% punishment trials (Figure 1). Since participants cannot predict reward or punishment based on their choice, a valid prediction about whether they will receive a reward or punishment could only be formed during the presentation of the cue. This design ensured that the number of trials per condition was equal in every participant. Two seconds after the choice of the expert was displayed, reward or punishment feedback was presented for 1,500 ms. A correct choice yielded +50 points; an incorrect choice yielded −40 points. Participants were instructed to obtain as many points as possible, since the participant with the highest score would receive additional monetary compensation. The aim of this instruction was for participants to attend to the actual outcome points rather than using the cues as feedback. The intertrial interval was jittered between 100 and 1,000 ms. A total of 300 trials was presented, with 120 congruent reward, 120 congruent punishment, 30 incongruent reward, and 30 incongruent punishment trials. The decision‐making task used in this experiment was programmed using Presentation software (Neurobehavioral Systems, Inc., Berkeley, CA).

### 2.3 EEG

EEG was recorded continuously during the task using an online 0.1–70 Hz band‐pass filter with a sampling rate of 1,000 Hz and 28 main electrodes: Fp1, Fp2, Fz, F3, F4, F7, F8, Fc1, Fc2, Fc5, Fc6, Cz, C3, C4, T7, T8, Cp1, Cp2, Cp5, Cp6, Pz, P3, P4, P7, P8, Oz, O1, and O2 (EASYCAP GmbH, Herrsching, Germany). The reference electrode was positioned over the left mastoid, and the ground electrode was placed at Fpz. Furthermore, a vertical electro‐oculogram (VEOG) was obtained by subtracting signal recordings from above and below the left eye. A horizontal electro‐oculogram (HEOG) was obtained by subtracting signal recordings from electrodes at the outer canthi of the eyes. All impedances were kept below 10 kΩ. Raw EEG data were recorded and stored for offline analysis using BrainVision Analyzer 2.1 (Brain Products GmbH, München, Germany).

### 2.4 Procedure

Participants were recruited using a campus database of healthy volunteers between 18 and 35 years of age. Initially, participants received information about the study and the EEG procedure, after which they filled out a safety screening, handedness form and provided written informed consent. Subsequently, participants were prepared for EEG recording in a comfortable chair that was placed ~80 cm in front of the computer screen. Before the beginning of the task, participants received written instructions and performed 10 practice trials. Then, participants performed the task, which lasted for approximately 30 min, with an intermediate break of 5 min. At the end, EEG equipment was removed, and participants received a monetary compensation of 10 Euros for their participation. The total duration of the experiment was approximately 1 hr.

### 2.5 Data reduction and analysis

Raw EEG signal recordings were offline band‐pass filtered between 0.1 and 30 Hz (48 dB/octave) and rereferenced to an average reference. VEOG and HEOG signals were used to correct for horizontal and vertical eye movement artifacts using the Gratton and Coles method (Gratton, Coles, & Donchin, 1983). The ERPs of interest were investigated at two different time points. First, ERPs were segmented time‐locked to expert cue presentation (prediction formation). In this analysis, two conditions were compared; namely, the expert cue agreed with the participant and a reward was anticipated (Exp+) versus the expert cue disagreed with the participant and a punishment was anticipated (Exp−). For this analysis, epochs started 100 ms before expert cue onset and ended 2,000 ms after expert cue onset (prediction information), which coincides with the time of feedback onset. Second, ERPs were segmented time‐locked to the moment participants received the feedback, which is when the prediction is evaluated. In this analysis, the task conditions (congruent‐reward/incongruent‐reward/congruent‐punishment/incongruent‐punishment) were compared, with epochs starting 100 ms before and ending 1,000 ms after feedback onset. For both analyses, a baseline correction with the window of −100 to 0 ms was used. Segments containing artifacts greater than 100 µV peak to peak were removed. Next, data were visually inspected for remaining non‐neurogenic sources of activity. For both analyses, the following averaged components were investigated: (a) the N1 at electrode Pz and Cz within a time window of 80–140 ms after cue/feedback onset (Doallo, Cadaveira, & Holguin, 2007; Ho et al., 2012); (b) the P2 at electrode Fz and Cz within a time window of 160–260 ms after cue/feedback onset (Wischnewski & Schutter, 2018); (c) the MFN at electrode Fz and Cz, which was determined as the difference between the first maximum and the subsequent minimum value in a time window of 150–350 ms after cue/feedback onset (Hajcak et al., 2005; Holroyd et al., 2003); and (d) the P3 at electrode Pz and Cz within a time window of 300–400 ms after cue/feedback onset (Balconi & Crivelli, 2010; Goyer, Woldorff, & Huettel, 2008). Additionally, the delay period until feedback onset was investigated at electrode Fz and Cz by calculating the mean amplitude within a time window of 600–2,000 ms after prediction formation. ERPs related to the onset of prediction formation will contain the prefix *p* (i.e., p‐N1, p‐P2, p‐MFN, p‐P3), whereas the components related to the onset of feedback processing will contain the prefix *f* (i.e., f‐N1, f‐P2, f‐MFN, f‐P3).

### 2.6 Statistical analysis

For the analysis of ERPs during prediction formation, a generalized linear model (GLM) repeated measures analysis of variance (ANOVA) for each ERP window and each electrode was used to compare the condition in a reward prediction (Exp+) and a punishment prediction (Exp−) was formed. For the analysis of ERPs during feedback processing, a 2 × 2 GLM within‐subject ANOVA was performed for each ERP window and each electrode with the factors valence (reward vs. punishment) and congruency (congruent vs. incongruent feedback). No correction was used for investigating multiple electrode locations. Finally, a multivariate linear regression was performed to test for the relationship between prediction formation and feedback processing signals. For this analysis, components showing a significant effect in the prediction formation analysis were entered as predictors. The valence and congruency effects of each component (f‐N1, f‐P2, f‐MFN, f‐P3) during feedback processing were entered as dependent variables. Significant effects were followed by Bonferroni‐corrected univariate linear regression analyses. All analyses were performed using IBM SPSS 22.0, and all statistical tests were compared to a two‐sided α significance level of .05.

## 3 RESULTS

### 3.1 ERP signals during prediction formation

**Figure 2:**
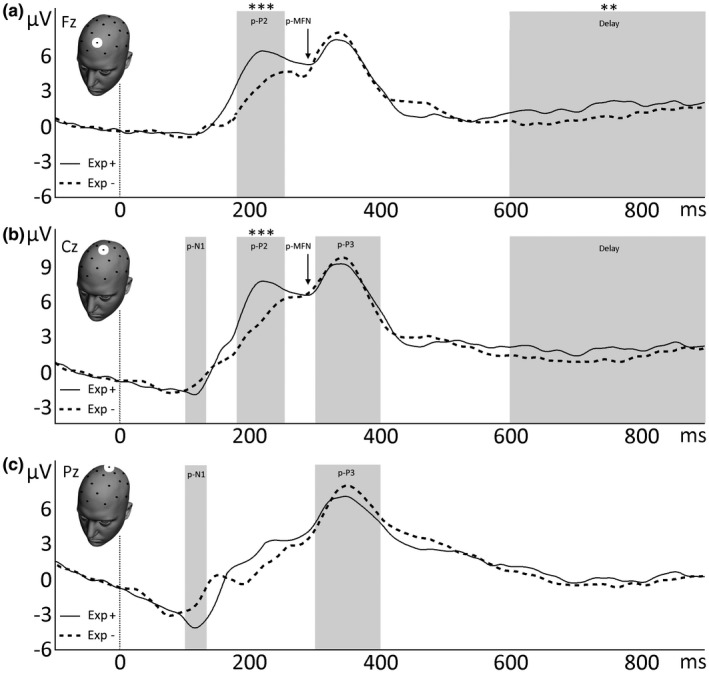
Grand‐averaged ERPs time‐locked to presentation of the expert cue, coinciding with the formation of an explicit prediction about future reward (Exp+, solid line) or punishment (Exp−, dotted line). ERPs from electrodes Fz (a), Cz (b) and Pz (c) are shown, and the investigated time windows are marked by a gray square or an arrow. Negativity is plotted downward. *p < .05; **p < .01; ***p < .001

A significant effect of prediction was observed in the p‐P2 component (mean amplitude 160–260 ms after expert cue) at electrode Fz, *F*(1, 19) = 25.57, *p* < .001, η_p_^2^ = .574 (Figure 2a) and Cz, *F*(1, 19) = 30.05, *p* < .001, η_p_^2^ = .613 (Figure 2b), with the prediction of reward (mean ± *SEM*, 5.72 ± 1.01 µV) yielding a larger p‐P2 amplitude than the prediction of punishment (mean ± *SEM*, 3.12 ± 0.86 µV). Furthermore, a significant prediction effect was observed in the delay period (mean amplitude 600–2,000 ms after expert cue) between expert cue and feedback in electrode Fz, *F*(1, 19) = 10.66, *p* = .004, η_p_^2^ = .359 (Figure 2a). The same trend was observed in electrode Cz, *F*(1, 19) = 4.27, *p* = .053, η_p_^2^ = .184 (Figure 2b). Within the delay period, a larger positivity was found on average for prediction of reward (mean ± *SEM*, 2.05 ± 0.38 µV) compared to prediction of punishment (mean ± *SEM*, 1.68 ± 0.34 µV). No significant prediction effect was observed for components p‐N1, p‐MFN, and p‐P3 (Figure 2, Table 1). To get a tentative idea of prediction‐related differences in oscillatory activity (Exp + vs. Exp−), a time‐frequency plot was created (see online supporting information, Figure S1). Visual inspection of the plot suggests that the difference between Exp+ and Exp− was accompanied by a difference in theta power (3–7 Hz) in the first 500 ms. During the delay period, 500 ms after the predictive cue until feedback onset, a difference in the 1–2 Hz (delta) frequency range was observed (Stefanics et al., 2010).

**Table 1:** T‐test results of prediction information (Exp+ vs. Exp−)

		*F*(1, 19)	*p*	η_p_^2^
p‐N1	Pz	1.33	.264	.065
	Cz	1.42	.248	.070
p‐P2	Fz	25.57	<.001***	.574
	Cz	30.05	<.001***	.613
p‐MFN	Fz	3.99	.060	.174
	Cz	0.97	.337	.049
p‐P3	Pz	1.19	.289	.059
	Cz	0.14	.714	.007
Delay	Fz	10.66	.004**	.359
	Cz	4.27	.053	.184

Abbreviation: MFN, medial frontal negativity.

** *p* < .01, ***p < .001.

### 3.2 ERP signals during feedback processing

**Figure 3:**
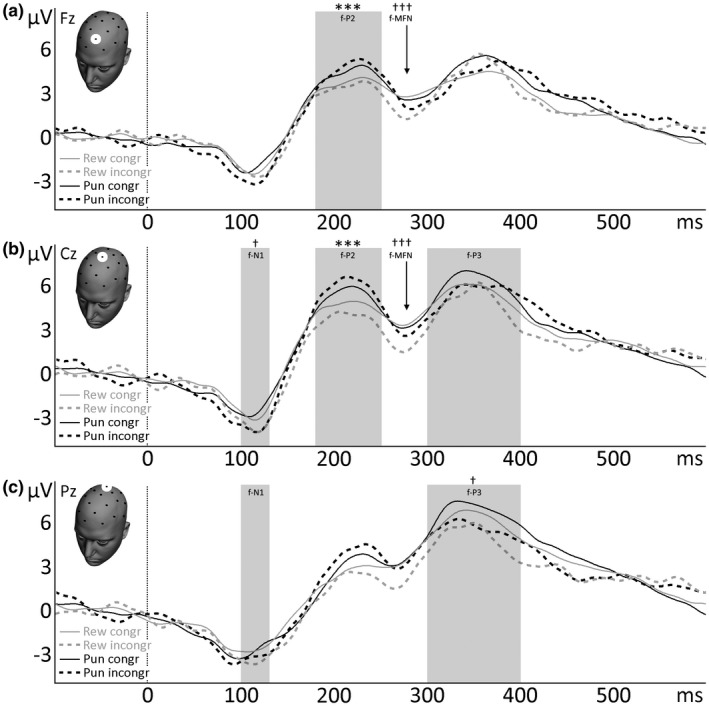
Grand‐averaged ERPs time‐locked to presentation of the feedback presentation, with four conditions: congruent reward (solid gray line), incongruent reward (dotted gray line), congruent punishment (solid black line), and incongruent punishment (dotted black line). ERPs from electrodes Fz (a), Cz (b) and Pz (c) are shown, and the investigated time windows are marked by a gray square or an arrow. Negativity is plotted downward. Asterisks indicate a significant effect of valence (reward vs. punishment), whereas obelisks indicate a significant effect of congruency (congruent vs. incongruent). *p < .05; **p < .01; ***p < .001

A significant effect of congruency was observed for f‐N1 (mean amplitude 80–140 ms after feedback) at the Cz electrode, *F*(1, 19) = 7.26, *p* = .014, η_p_^2^ = .276 (Figure 3b), indicating that incongruent feedback was related to a larger negative amplitude (mean ± *SEM*, −3.72 ± 0.62 µV) as compared to congruent feedback (−2.79 ± 0.51 µV). Neither a significant main effect of valence nor a Congruency × Valence interaction effect was observed for f‐N1 (Table 2, Figure 3). For the f‐P2 (mean amplitude 160–260 ms after feedback) component, a significant effect of valence was observed in electrode Fz, *F*(1, 19) = 12.04, *p* = .003, η_p_^2^ = .388 (Figure 3a) and Cz, *F*(1, 19) = 28.09, *p* < .001, η_p_^2^ = .597 (Figure 3b). Negative feedback (mean ± *SEM*, 4.42 ± 0.61 µV) elicited larger f‐P2 amplitudes than positive feedback (mean ± *SEM*, 3.47 ± 0.57 µV). No significant effect of congruency and Congruency × Valence were observed for f‐P2 (Table 2). Analysis on the f‐MFN component (maximum‐minimum difference between 150–350 ms after feedback) showed a significant main effect of congruency at the Fz electrode, *F*(1, 19) = 23.35, *p* < .001, η_p_^2^ = .551 (Figure 3a) and Cz, *F*(1, 19) = 12.90, *p* = .002, η_p_^2^ = .404 (Figure 3b). Incongruent feedback elicited larger f‐MFN amplitudes (mean ± *SEM*, −5.97 ± 0.64 µV) compared to congruent feedback (mean ± *SEM*, −4.53 ± 0.39 µV). No significant effects of valence and Congruency × Valence were observed (Table 2). For the f‐P3 amplitude (mean amplitude 300–400 ms after feedback), a significant effect of congruency was observed in Pz, *F*(1, 19) = 6.33, *p* = .021, η_p_^2^ = .250 (Figure 3c). Congruent trials (mean ± *SEM*, 6.43 ± 0.71 µV) elicited a larger f‐P3 than incongruent trials (mean ± *SEM*, 5.29 ± 0.59 µV). No significant effects of valence and Congruency × Valence were observed (Table 2).

**Table 2:** GLM repeated measures ANOVA results of valence (reward vs. punishment) and congruency (congruent vs. incongruent) effects during feedback processing

		Valence			Congruency			Valence × Congruency		
		*F*(1, 19)	*p*	η_p_^2^	*F*(1, 19)	*p*	η_p_^2^	*F*(1, 19)	*p*	η_p_^2^
f‐N1	Pz	0.45	.508	.023	3.12	.094	.141	0.48	.499	.025
	Cz	0.21	.653	.011	7.26	.014†	.276	0.07	.789	.004
f‐P2	Fz	12.04	.003**	.388	<0.01	.985	<.001	0.39	.541	.020
	Cz	28.09	<.001***	.597	0.04	.839	.002	2.87	.107	.131
f‐MFN	Fz	1.63	.217	.079	23.35	<.001†††	.551	0.12	.733	.006
	Cz	4.25	.053	.183	12.90	.002††	.404	<0.01	.996	<.001
f‐P3	Pz	1.41	.250	.069	6.33	.021†	.250	0.14	.713	.007
	Cz	1.23	.282	.061	1.09	.309	.054	0.10	.751	.005

Note.

Asterisk refers to an effect of valence. Dagger refers to an effect of congruency. Abbreviation: MFN, medial frontal negativity.

*,† *p* < .05. **^,††^ *p *< .01. ***^,†††^ *p* < .001.

### 3.3 Relationship between prediction formation and feedback processing signals

**Figure 4:**
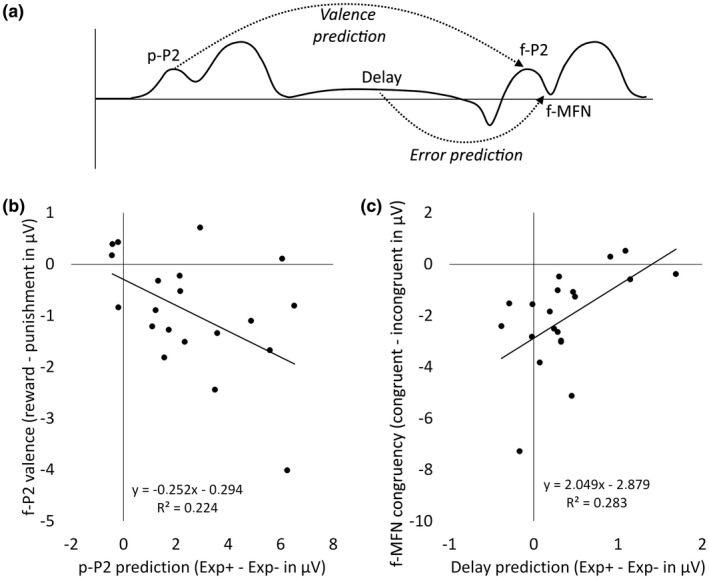
(a) Schematic representation of the multivariate regression analysis results. (b) Signal difference in p‐P2 (Exp + minus Exp−) was significantly inversely correlated to the valence effect (reward minus punishment) in subsequent f‐P2. (c) Signal difference in p‐P2 (Exp + minus Exp−) was significantly positively correlated to the congruency effect (congruent minus incongruent) in subsequent f‐MFN

The prediction effect of p‐P2 and the delay period (difference between Exp+ and Exp−) were entered as predictors in a multivariate linear regression analysis. Results showed that the p‐P2 is a significant predictor for subsequent feedback processing signals, *F*(4, 14) = 3.45, *p* = .037. Bonferroni‐corrected post hoc univariate linear regressions showed the p‐P2 difference as a significant predictor for the f‐P2 valence effect, *F*(1, 17) = 11.84, *p* = .025 (Figure 4b). Valence and congruency effects in other ERP components were not associated with p‐P2 (*p*s > .24). Together, these results provide evidence that the p‐P2 is a predictive signal for subsequent reward and punishment processing in f‐P2 (Figure 4a). Furthermore, the multivariate regression analysis showed that delay period prediction effect is a significant predictor for subsequent feedback processing signals, *F*(4, 14) = 4.14, *p* = .020. Bonferroni‐corrected post hoc univariate linear regressions showed the delay difference as a significant factor for the congruency effect of p‐MFN, *F*(1, 17) = 13.91, *p* = .013 (Figure 4c). These results imply that the delay period reflects a predictive signal for subsequent feedback‐related error processing (Figure 4a). Valence and congruency effects in the other ERP components were not predicted by the delay period (*p*s > .18).

## 4 DISCUSSION

The present study explored the electrophysiological correlates of prediction formation and how these predictions affect subsequent feedback processing. During feedback processing, the f‐P2 component was found to be larger for punishments compared to rewards. Although a valence effect on the P2 amplitude has been repeatedly shown by previous studies, results on the directionality of this effect have so far been inconsistent (Bellebaum, Kobza, Thiele, & Daum, 2010; Polezzi, Lotto, Daum, Sartori, & Rumiati, 2008; San Martin, Manes, Hurtado, Isla, & Ibañez, 2010; Schuermann, Endrass, & Kathmann, 2012; Xu et al., 2011). While some studies found larger P2 amplitudes for reward in comparison to punishment feedback (Bellebaum et al., 2010; San Martin et al., 2010; Xu et al., 2011), others have found larger P2 amplitudes to punishment‐ compared to reward‐related feedback (Carretié, Hinojosa, Mercado, & Tapia, 2005; Carretié, Mercado, Tapia, & Hinojosa, 2001; Polezzi et al., 2008, Schuermann et al., 2012). In a previous study, we provided evidence that these contrasting results may be a consequence of context. In a context where alternative outcomes are shown, reward was associated with larger P2 amplitudes compared to punishment (Wischnewski & Schutter, 2018). However, when no alternative outcomes were shown, rewards were associated with smaller P2 amplitudes compared to punishments during feedback processing (Wischnewski & Schutter, 2018). As in the latter case, in the present study no alternative outcomes were shown, and increased P2 amplitudes were expected for the anticipation of reward (p‐P2) and reward feedback (f‐P2). Surprisingly, a negative rather than a positive relationship between p‐P2 and f‐P2 was found. The anticipation of reward yielded larger p‐P2 amplitudes than anticipation of punishment, in line with our previous findings (Wischnewski & Schutter, 2018). However, the directionality of valence was inversed during feedback presentation, where reward feedback yielded smaller f‐P2 amplitudes than punishment feedback. Since the P2 has been suggested to be related to attentional processes, these results may suggest that more attention is allocated to information that predicts a reward rather than a punishment. When a reward is subsequently received, no additional increase in attention is prompted. Since attention was not actively manipulated in the present study, this interpretation remains speculative. However, these findings seem analogous to reports in nonhuman primates where dopamine release from the ventral tegmental area to the striatum is related to the prediction of reward, with no further dopamine release being observed when actually receiving this reward (Schultz, 2007; Schultz, Dayan, & Montague, 1997). Indeed, the P2 may reflect a similar process, since this component is proposed to be associated with dopaminergic activity in the mesocorticolimbic tract (Gui, Li, Li, & Luo, 2016; Kiat, Straley, & Cheadle, 2016; Morie, De Sanctis, & Foxe, 2014; Proudfit, 2015). That is, when the expert cue predicts a forthcoming reward, dopamine is released. This dopamine release coincides with the direction of attentional resources toward the cue, reflected by the P2. During actual feedback, when the reward is received, no dopamine release occurs and thus no attention is directed toward this reward, arguably yielding smaller P2 amplitudes. Ultimately, from the present and our previous study (Wischnewski & Schutter, 2018), it has become apparent that valence effects in P2 depend on a variety of contextual factors, and more research is needed to fully understand the mechanisms underlying this component.

In agreement with earlier findings, a significant congruency effect was found in the f‐MFN (Alexander & Brown, 2011; Hajcak et al., 2005, 2007; Holroyd et al., 2003, 2004; Weismüller & Bellebaum, 2016). Incongruent feedback was associated with increased negativity (i.e., large prediction error), compared to congruent feedback, which yielded reduced negativity (i.e., small prediction error). The absence of a valence and interaction effect indicated that this increased negativity was seen for both reward‐ and punishment‐related prediction errors. Currently, it is debated whether the MFN reflects a reward prediction error (Bellebaum, Polezzi, & Daum, 2010; Hajcak et al., 2005, 2007; Holroyd et al., 2003) or general mismatch detection (Alexander & Brown, 2011; Jessup et al., 2010; Talmi et al., 2013). Our results provide evidence for the latter hypothesis. Although no distinction between reward and punishment prediction error was observed in the f‐MFN component, a difference in signals of reward and punishment prediction was observed in the delay period before feedback onset. This difference in the delay period was positively correlated to the discrepancy between mismatches and nonmismatches of predicted and actual outcome in the f‐MFN (Figure 4). The relationship between prediction and feedback signals suggests that, although no valence effect was found during feedback processing (f‐MFN), dissociable signals for reward and punishment were present during anticipation in the delay period. Furthermore, the positive relationship between prediction in the delay period and error signals in the f‐MFN suggests that the more divergent prediction signals are, the more distinctive the signals for error processing are. Slow wave activity in the delta band, as observed during the delay period (supporting information, Figure S1), has been related to heightened selective attention (Lakatos, Karmos, Mehta, Ulbert, & Schroeder, 2008; Stefanics et al., 2010). It has been proposed that this selective attention enhances detection and discriminability between various stimuli (Schroeder & Lakatos, 2009; Summerfield & Egner, 2009). Selective attention may therefore play a role in the future detection of mismatches in predicted and actual reward and punishments.

Whereas a slow‐wave difference between following and not following the expert cue was observed during the delay period, no increasing negativity (i.e., SPN) was observed. It could be speculated that the absence of the SPN may be explained by high probability of the correctness of the expert cue. The cue already contains information on both the correctness of the response as well as upcoming reward or punishment. As such, there is no strong anticipation for the correctness and valence of the outcome, and therefore no SPN is processed. Instead, participants may anticipate whether the expert cue was correct or wrong, thus reflecting anticipation of an error in prediction. This may explain why no reflection of valence prediction was observed within the delay period. To confirm this speculation, however, a similar experiment needs to be repeated in which valence of outcome is manipulated more systematically.

The present results also indicated that the early attention component N1 does reflect an effect of mismatch detection. This suggests that the outcome of the comparison between anticipated and actual outcome is already detected at an early stage of feedback processing. Trautman‐Lengsfeld and Herrmann (2013) have proposed that such early attention components can be biased by the outcome prediction, due to differential processing of mismatches and nonmismatches in visual areas. However, we did not find a relationship between prediction information signals and N1 feedback processing component. The N1 component is thought to reflect a visual sensory process influenced by subcortical regions including the amygdala (Shackman, Maxwell, McMenamin, Greischar, & Davidson, 2011). Indeed, N1 amplitude is shown to be increased in trials with increased vigilance and stress, particularly in response to aversive stimuli (Doallo et al., 2007; Shackman et al., 2011 You & Li, 2016). On a speculative account, the congruency effect of N1 observed in the present study may reflect increased vigilance toward the detection of unexpected outcomes and may prime subsequent attention allocation as reflected by the P3 (Shackman et al., 2011). Unfortunately, N1 is not regularly investigated in performance monitoring studies, and thus more evidence from future studies is needed to test this hypothesis.

The difference between mismatch and nonmismatch of predicted and actual outcome (i.e., the congruency effect) during feedback signals was observed for the P3 component, which is in agreement with previous studies (Bellebaum & Daum, 2008; Hajcak et al., 2005; Pfabigan, Alexopoulos, Bauer, & Sailer, 2011). However, this effect was not related to the studied ERP components during prediction formation and the subsequent delay period. P3 has been suggested to index an endogenous process of attentional allocation related to surprise (Polich, 2007). This explains the difference in P3 signal between trials with and without a prediction error (Fischer & Ullsperger, 2013; Pfabigan et al., 2011). As such, attention allocation is a consequence of prediction error processing, as indexed by the MFN, in which potentially action‐relevant situations are highlighted (Ullsperger, Fischer, Nigbur, & Endrass, 2014). Therefore, since the attentional allocation of the P3 depends on error processing (reflected by the MFN), the effects on P3 observed here may reflect an ad hoc phenomenon. This suggestion would imply that if no effect of congruency is observed in MFN, neither will there be a demand for attentional allocation, as indexed by the P3. Still, the lack of a valence effect on the P3 is notable. Even though this result contrasts studies that did observe an effect of valence on P3 amplitude (Bellebaum, Polezzi, & Daum, 2010; Flores, Münte, & Donamayor, 2015; Kreussel et al., 2012; Schevernels, Krebs, Santens, Woldorff, & Boehler, 2014), others have reported an absence of valence effects on P3 (Goyer et al., 2008; Sato et al., 2005; Von Borries, Verkes, Bulten, Cools, & de Bruijn 2013; Yeung & Sanfey, 2004). It is conceivable that these valence specific effects depend on the opportunity to learn in a task, since the attention allocation processes associated with P3 are relevant for error minimization and learning (Fischer & Ullsperger, 2013; Ullsperger et al., 2014). Although the present task demonstrated a mismatch between predicted and actual outcome, this information could not be used by the participants for error minimization. Processing of valence in feedback signals is necessary for learning, since a punishment would require a change of action in the future, whereas a reward would require the same action to be repeated. It is possible that the absence of learning effects, due to pseudorandom feedback, attenuates distinct reward and punishment processing reflected by P3 (Sato et al., 2005; Yeung & Sanfey, 2004).

In conclusion, the present study offers novel insights in the electrophysiological components related to prediction formation before feedback processing. The evaluation of mismatch or nonmismatch between predicted and actual outcome (i.e., prediction errors) and reward or punishment (i.e., valence) are both reflected by signal differences before actual feedback processing. First, the P2 component during prediction formation encodes expectancy of future rewards and punishments. Second, the delay period, which encodes expectancy of future errors in prediction, is possibly related to selective attention toward unexpected outcomes.

## Supporting information


**Figure S1**

## References

[psyp13379-bib-0001] Alexander, W. H. , & Brown, J. W. (2011). Medial prefrontal cortex as an action‐outcome predictor. Nature Neuroscience, 14, 1338–1344. 10.1038/nn.2921 21926982 PMC3183374

[psyp13379-bib-0002] Alexander, W. H. , & Brown, J. W. (2018). Frontal cortex function as derived from hierarchical predictive coding. Science Reports, 8, 3843 10.1038/s41598-018-21407-9 PMC5832795 29497060

[psyp13379-bib-0004] Balconi, M. , & Crivelli, D. (2010). FRN and P300 ERP effect modulation in response to feedback sensitivity: The contribution of punishment‐reward system (BIS/BAS) and behaviour identification of action. Neuroscience Research, 66(2), 162–172. 10.1016/j.neures.2009.10.011 19895858

[psyp13379-bib-0005] Bellebaum, C. , & Daum, I. (2008). Learning‐related changes in reward expectancy are reflected in the feedback‐related negativity. European Journal of Neuroscience, 27, 1823–1835. 10.1111/j.1460-9568.2008.06138.x 18380674

[psyp13379-bib-0006] Bellebaum, C. , Kobza, S. , Thiele, S. , & Daum, I. (2010). It was not MY fault: Event‐related brain potentials in active and observational learning from feedback. Cerebral Cortex, 20(12), 2874–2883. 10.1093/cercor/bhq038 20308202

[psyp13379-bib-0007] Bellebaum, C. , Polezzi, D. , & Daum, I. (2010). It is less than you expected: The feedback‐related negativity reflects violations of reward magnitude expectations. Neuropsychologia, 48(11), 3343–3350. 10.1016/j.neuropsychologia.2010.07.023 20655319

[psyp13379-bib-0008] Bengson, J. J. , Kelley, T. A. , & Mangun, G. R. (2015). The neural correlates of volitional attention: A combined fMRI and ERP study. Human Brain Mapping, 36, 2443–2454. 10.1002/hbm.22783 25731128 PMC6869709

[psyp13379-bib-0009] Brunia, C. M. H. , van Boxtel, G. J. M. , & Böcker, K. B. E. (2012). Negative slow waves as indices of anticipation: The bereitshaftspotential, the contingent negative variation, and the stimulus preceding negativity In Luck S. J. &Kappenman E. (Eds.), Oxford handbook of event‐related potential components. New York, NY: Oxford University Press 10.1093/oxfordhb/9780195374148.013.0108

[psyp13379-bib-0010] Carretié, L. , Hinojosa, J. A. , Mercado, J. A. F. , & Tapia, M. (2005). Cortical response to subjectively unconscious danger. NeuroImage, 24, 615–623. 10.1016/j.neuroimage.2004.09.009 15652297

[psyp13379-bib-0011] Carretié, L. , Mercado, J. A. F. , Tapia, M. , & Hinojosa, J. A. (2001). Emotion, attention, and the ‘negativity bias’, studied through event‐related potentials. International Journal of Psychophysiology, 41, 75–85. 10.1016/S0167-8760(00)00195-1 11239699

[psyp13379-bib-0012] Doallo, S. , Cadaveira, F. , & Holguin, S. R. (2007). Time course of attentional modulations of automatic emotional processing. Neuroscience Letters, 418(1), 111–116. 10.1016/j.neulet.2007.03.009 17399898

[psyp13379-bib-0013] Fischer, A. G. , & Ullsperger, M. (2013). Real and fictive outcomes are processed differently but converge on a common adaptive mechanisms. Neuron, 79(6), 1243–1255. 10.1016/j.neuron.2013.07.006 24050408

[psyp13379-bib-0014] Flores, A. , Münte, T. F. , & Donamayor, N. (2015). Event‐related EEG responses to anticipation and delivery of monetary and social reward. Biological Psychology, 109, 10–19. 10.1016/j.biopsycho.2015.04.005 25910956

[psyp13379-bib-0015] Friston, K. (2005). A theory of cortical responses. Philosophical Transactions of the Royal Society of London B: Biological Science, 360(1456), 815–836. 10.1098/rstb.2005.1622 PMC1569488 15937014

[psyp13379-bib-0016] Friston, K. , & Kiebel, S. (2009). Predictive coding under the free‐energy principle. Philosophical Transactions of the Royal Society B, 364, 1211–1221. 10.1098/rstb.2008.0300 PMC2666703 19528002

[psyp13379-bib-0017] Goyer, J. P. , Woldorff, M. G. , & Huettel, S. A. (2008). Rapid electrophysiological brain responses are influenced by both valence and magnitude of monetary rewards. Journal of Cognitive Neuroscience, 20(11), 2058–2069. 10.1162/jocn.2008.20134 18416673 PMC3612129

[psyp13379-bib-0018] Gratton, G. , Coles, M. G. , & Donchin, E. (1983). A new method for off‐line removal of ocular artifacts. Electroencephalography and Clinical Neurophysiology, 55(4), 468–484. 10.1016/0013-4694(83)90135-9 6187540

[psyp13379-bib-0019] Gui, D. Y. , Li, J. Z. , Li, X. , & Luo, Y. J. (2016). Temporal dynamics of the interaction between reward and time delay during intertemporal choice. Frontiers in Psychology, 7, 1526 10.3389/fpsyg.2016.01526 27785126 PMC5060948

[psyp13379-bib-0020] Hajcak, G. , Holroyd, C. B. , Moser, J. S. , & Simons, R. F. (2005). Brain potentials associated with expected and unexpected good and bad outcomes. Psychophysiology, 42, 161–170. 10.1111/j.1469-8986.2005.00278.x 15787853

[psyp13379-bib-0021] Hajcak, G. , Moser, J. S. , Holroyd, C. B. , & Simons, R. F. (2007). It's worse than you thought: The feedback negativity and violations of reward prediction in gambling tasks. Psychophsyiology, 44(6), 905–912. 10.1111/j.1469-8986.2007.00567.x 17666029

[psyp13379-bib-0022] Ho, M. C. , Chou, C. Y. , Huang, C. F. , Lin, Y. T. , Shih, C. S. , Han, S. Y. , … Liu, C. J. (2012). Age‐related changes in task‐specific brain activity in normal aging. Neuroscience Letters, 507(1), 78–83. 10.1016/j.neulet.2011.11.057 22172937

[psyp13379-bib-0023] Holroyd, C. B. , Larsen, J. T. , & Cohen, J. D. (2004). Context dependence of the event‐related brain potential associated with reward and punishment. Psychophysiology, 41, 245–253. 10.1111/j.1469-8986.2004.00152.x 15032989

[psyp13379-bib-0024] Holroyd, C. B. , Nieuwenhuis, S. , Yeung, N. , & Cohen, J. D. (2003). Errors in reward prediction are reflected in the event‐related brain potential. NeuroReport, 14(18), 2481–2484. 10.1097/01.wnr.0000099601.41403.a5 14663214

[psyp13379-bib-0025] Jessup, R. K. , Busemeyer, J. R. , & Brown, J. W. (2010). Error effects in anterior cingulate cortex reverse when error likelihood is high. Journal of Neuroscience, 30(9), 3467–3472. 10.1523/JNEUROSCI.4130-09.2010 20203206 PMC2841347

[psyp13379-bib-0026] Kahneman, D. , & Tversky, A. (1979). Prospect theory: An analysis of decision under risk. Econometrica, 47(2), 263–291. 10.2307/1914185

[psyp13379-bib-0027] Kiat, J. , Straley, E. , & Cheadle, J. E. (2016). Escalating risk and the moderating effect of resistance to peer influence on the P200 and feedback‐related negativity. Social, Cognitive, and Affective Neuroscience, 11(3), 377–386. 10.1093/scan/nsv121 26416785 PMC4769624

[psyp13379-bib-0028] Kreussel, L. , Hewig, J. , Kretschmer, N. , Hecht, H. , Coles, M. G. H. , & Miltner, W. H. R. (2012). The influence of the magnitude, probability, and valence of potential wins and losses on the amplitude of feedback negativity. Psychophysiology, 49, 207–219. 10.1111/j.1469-8986.2011.01291.x 22091824

[psyp13379-bib-0029] Lakatos, P. , Karmos, G. , Mehta, A. D. , Ulbert, I. , & Schroeder, C. E. (2008). Entrainment of neuronal oscillations as a mechanism of attention selection. Science, 320, 110–113. 10.1126/science.1154735 18388295

[psyp13379-bib-0030] Meshi, D. , Biele, G. , Korn, C. W. , & Heekeren, H. R. (2012). How expert advice influences decision making. PLOS One, 7(11), e49748 10.1371/journal.pone.0049748 23185425 PMC3504100

[psyp13379-bib-0031] Morie, K. P. , De Sanctis, P. , & Foxe, J. J. (2014). Reward contingencies and the recalibration of task monitoring and reward systems: A high‐density electrical mapping study. Neuroscience, 273, 100–117. 10.1016/j.neuroscience.2014.05.002 24836852 PMC4209734

[psyp13379-bib-0032] Oldfield, R. (1971). The assessment and analysis of handedness: The Edinburgh Inventory. Neuropsychologia, 9(1), 97–113. 10.1016/0028-3932(71)90067-4 5146491

[psyp13379-bib-0033] Pfabigan, D. M. , Alexopoulos, J. , Bauer, H. , & Sailer, U. (2011). Manipulation of feedback expectancy and valence induces negative and positive reward prediction error signals manifest in event‐related brain potentials. Psychophysiology, 48(5), 656–664. 10.1111/j.1469-8986.2010.01136.x 21039585

[psyp13379-bib-0034] Pfabigan, D. M. , Seidel, E. M. , Sladky, R. , Hahn, A. , Paul, K. , Grahl, A. , … Lamm, C. (2014). P300 amplitude variation is related to ventral striatum BOLD response during gain and loss anticipation: An EEG and fMRI experiment. NeuroImage, 96, 12–21. 10.1016/j.neuroimage.2014.03.077 24718288 PMC4075343

[psyp13379-bib-0035] Polezzi, D. , Lotto, L. , Daum, I. , Sartori, G. , & Rumiati, R. (2008). Predicting outcomes of decisions in the brain. Behavioural Brain Research, 187, 116–122. 10.1016/j.bbr.2007.09.001 17935798

[psyp13379-bib-0036] Polich, J. (2007). Updating P300: An integrative theory of P3a and P3b. Clinical Neurophysiology, 118(10), 2128–2148. 10.1016/j.clinph.2007.04.019 17573239 PMC2715154

[psyp13379-bib-0037] Proudfit, G. H. (2015). The reward positivity: From basic research on reward to a biomarker for depression. Psychophysiology, 52(4), 449–459. 10.1111/psyp.12370 25327938

[psyp13379-bib-0038] Rushworth, M. F. S. , Mars, R. B. , & Summerfield, C. (2009). General mechanisms for making decisions? Current Opinions in Neurobiology, 19(1), 75–83. 10.1016/j.conb.2009.02.005 19349160

[psyp13379-bib-0039] San Martin, R. , Manes, F. , Hurtado, E. , Isla, P. , & Ibañez, A. (2010). Size and probability of rewards modulate the feedback error‐related negativity associated with wins but not losses in a monetarily rewarded gambling task. NeuroImage, 51, 1194–1204. 10.1016/j.neuroimage.2010.03.031 20302950

[psyp13379-bib-0040] Sato, A. , Yasuda, A. , Ohira, H. , Miyawaki, K. , Nishikawa, M. , Kumano, H. , … Kuboki, T. (2005). Effects of value and reward magnitude on feedback negativity and P300. NeuroReport, 16(4), 407–411. 10.1097/00001756-200503150-00020 15729147

[psyp13379-bib-0041] Schevernels, H. , Krebs, R. M. , Santens, P. , Woldorff, M. G. , & Boehler, C. N. (2014). Task preparation processes related to reward prediction precede those related to task‐difficulty expectation. NeuroImage, 84, 639–647. 10.1016/j.neuroimage.2013.09.039 24064071 PMC3863725

[psyp13379-bib-0042] Schroeder, C. E. , & Lakatos, P. (2009). Low‐frequency neuronal oscillations as instruments of sensory selection. Trends in Neuroscience, 32, 9–18. 10.1016/j.tins.2008.09.012 PMC2990947 19012975

[psyp13379-bib-0043] Schuermann, B. , Endrass, T. , & Kathmann, N. (2012). Neural correlates of feedback processing in decision‐making under risk. Frontiers in Human Neuroscience, 6, 204 10.3389/fnhum.2012.00204 22783182 PMC3390593

[psyp13379-bib-0044] Schultz, W. (2007). Behavioral dopamine signals. Trends in Neuroscience, 30(5), 203–210. 10.1016/j.tins.2007.03.007 17400301

[psyp13379-bib-0045] Schultz, W. , Dayan, P. , & Montague, P. R. (1997). A neural substrate of prediction and reward. Science, 275, 1593–1599. 10.1126/science.275.5306.1593 9054347

[psyp13379-bib-0046] Shackman, A. J. , Maxwell, J. S. , McMenamin, B. W. , Greischar, L. L. , & Davidson, R. J. (2011). Stress potentiates early and attenuates late stages of visual processing. Journal of Neuroscience, 31(3), 1156–1161. 10.1523/JNEUROSCI.3384-10.2011 21248140 PMC3037336

[psyp13379-bib-0047] Stefanics, G. , Hangya, B. , Hernadi, I. , Winkler, I. , Lakatos, P. , & Ulbert, I. (2010). Phase entrainment of human delta oscillations can mediate the effects of expectation on reaction speed. Journal of Neuroscience, 30(41), 13578–13585. 10.1523/JNEUROSCI.0703-10.2010 20943899 PMC4427664

[psyp13379-bib-0048] Summerfield, C. , & Egner, T. (2009). Expectation (and attention) in visual cognition. Trends in Cognitive Science, 13(9), 403–409. 10.1016/j.tics.2009.06.003 19716752

[psyp13379-bib-0049] Summerfield, C. , Egner, T. , Greene, M. , Koechlin, E. , Mangels, J. , & Hirsch, J. (2006). Predictive codes for forthcoming perception in the frontal cortex. Science, 314(5803), 1311–1314. 10.1126/science.1132028 17124325

[psyp13379-bib-0050] Talmi, D. , Atkinson, R. , & El‐Deredy, W. (2013). The feedback‐related negativity signals salience prediction errors not reward prediction errors. Journal of Neuroscience, 33(19), 8264–8269. 10.1523/JNEUROSCI.5695-12.2013 23658166 PMC6619637

[psyp13379-bib-0051] Trautmann‐Lengsfeld, S. A. , & Herrmann, C. S. (2013). EEG reveals an early influence of social conformity on visual processing in group pressure situations. Social Neuroscience, 8(1), 75–89. 10.1080/17470919.2012.742927 23163969

[psyp13379-bib-0052] Tversky, A. , & Kahneman, D. (1992). Advances in prospect theory: Cumulative representation of uncertainty. Journal of Risk and Uncertainty, 5, 297–323. 10.1007/978-3-319-20451-2_24

[psyp13379-bib-0053] Ullsperger, M. , Fischer, A. G. , Nigbur, R. , & Endrass, T. (2014). Neural mechanisms and temporal dynamics of performance monitoring. Trends in Cognitive Science, 18(5), 259–267. 10.1016/j.tics.2014.02.009 24656460

[psyp13379-bib-0054] Van Boxtel, G. J. M. , & Böcker, K. B. E. (2004). Cortical measures of anticipation. Journal of Psychophysiology, 18, 61–76. 10.1027/0269-8803.18.23.61

[psyp13379-bib-0055] Van Pelt, S. , Heil, L. , Kwisthout, J. , Ondobaka, S. , Van Rooij, I. , & Bekkering, H. (2016). Beta‐ and gamma‐band activity reflect predictive coding in the processing of causal events. Social, Cognitive, and Affective Neuroscience, 11(6), 973–980. 10.1093/scan/nsw017 26873806 PMC4884316

[psyp13379-bib-0056] Von Borries, A. K. L. , Verkes, R. J. , Bulten, B. H. , Cools, R. , & de Bruijn, E. R. A. (2013). Feedback‐related negativity codes outcome valence, but not outcome expectancy, during reversal learning. Cognitive, Affective, and Behavioral Neuroscience, 13(4), 737–746. 10.3758/s13415-013-0150-1 24146314

[psyp13379-bib-0057] Weismüller, B. , & Bellebaum, C. (2016). Expectancy affects the feedback‐related negativity (FRN) for delayed feedback in probabilistic learning. Psychophysiology, 53(11), 1739–1750. 10.1111/psyp.12738 27565454

[psyp13379-bib-0058] Wischnewski, M. , Bekkering, H. , & Schutter, D. J. L. G. (2018). Frontal cortex electrophysiology in reward‐ and punishment‐related feedback processing during advice‐guided decision making: An interleaved EEG‐DC stimulation study. Cognitive, Affective, and Behavioral Neuroscience, 18(2), 249–262. 10.3758/s13415-018-0566-8 PMC5889418 29380293

[psyp13379-bib-0059] Wischnewski, M. , & Schutter, D. J. L. G. (2017). After‐effects of transcranial alternating current stimulation on evoked delta and theta power. Clinical Neurophysiology, 128(11), 2227–2232. 10.1016/j.clinph.2017.08.029 28987994

[psyp13379-bib-0060] Wischnewski, M. , & Schutter, D. J. L. G. (2018). Dissociating absolute and relative reward‐ and punishment‐related electrocortical processing: An event‐related potential study. Internationnal Journal of Psychophysiology, 126, 13–19. 10.1016/j.ijpsycho.2018.02.010 29481828

[psyp13379-bib-0061] Xu, Q. , Shen, Q. , Chen, P. , Ma, Q. , Sun, D. , & Pan, Y. (2011). How an uncertain cue modulates subsequent monetary outcome evaluation: An ERP study. Neuroscience Letters, 505(2), 200–204. 10.1016/j.neulet.2011.10.024 22027182

[psyp13379-bib-0062] Yeung, N. , & Sanfey, A. G. (2004). Independent coding of reward magnitude and valence in the human brain. Journal of Neuroscience, 24(28), 6258–6264. 10.1523/JNEUROSCI.4537-03.2004 15254080 PMC6729539

[psyp13379-bib-0063] You, Y. , & Li, W. (2016). Parallel processing of general and specific threat during early stages of perception. Social, Cognitive, and Affective Neuroscience, 11(3), 395–404. 10.1093/scan/nsv123 26412811 PMC4769625

