# Supplementary material for: Electrophysiological correlates of prediction formation in anticipation of reward‐ and punishment‐related feedback signals
Source: Psychophysiology. 2019 Apr 26;56(8):e13379. doi: 10.1111/psyp.13379 (PMC6850464; doi:10.1111/psyp.13379)
Supplement: Supplementary file 1 — Figure S1 [file PSYP-56-na-s001.docx]

**Supplementary data**


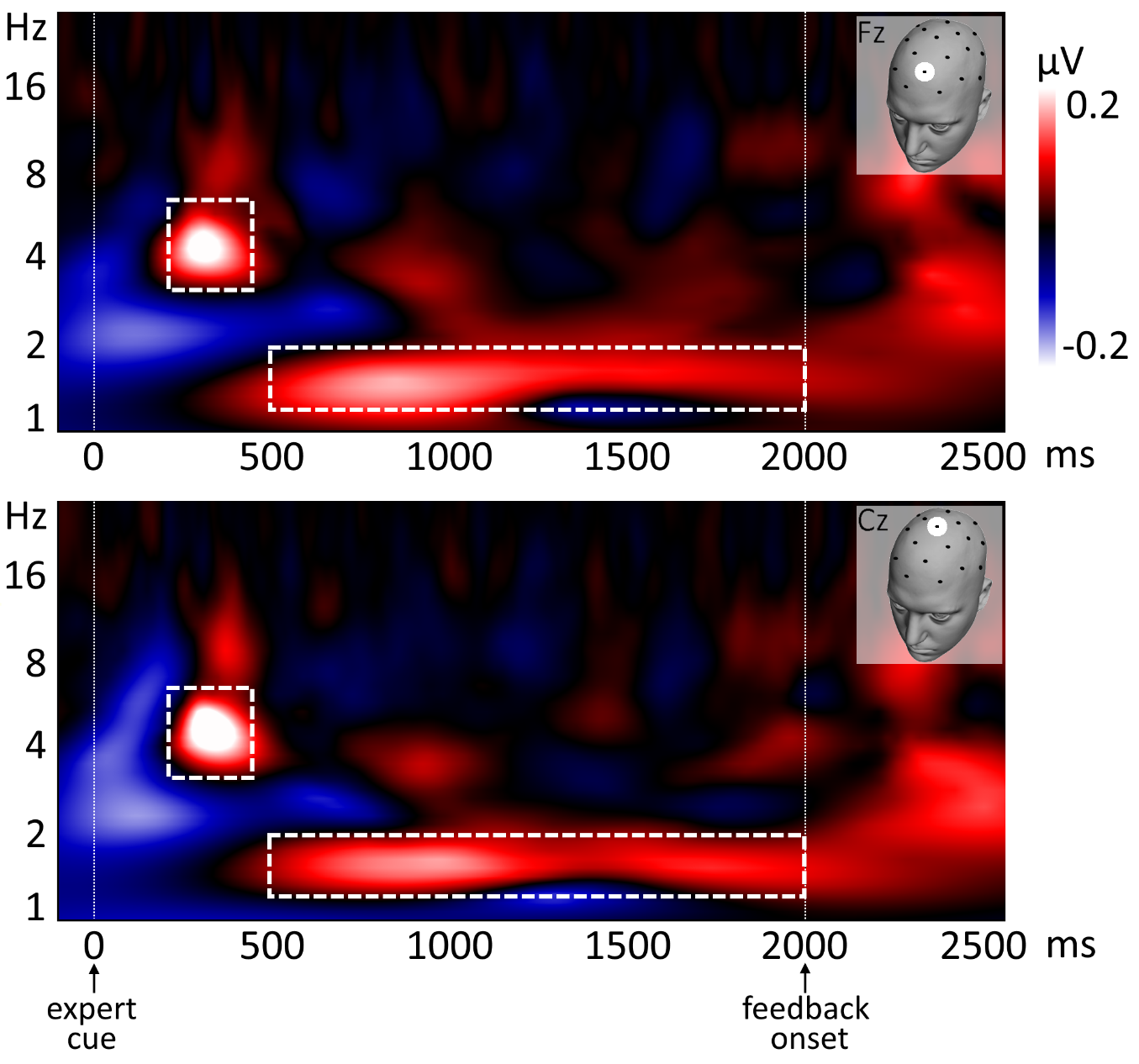


Figure S1. To get an idea of prediction-related differences in oscillatory activity (Exp+ vs Exp-), a time-frequency plot was created. Fast Fourier transformed power spectra were analyzed using complex Morlet wavelets, with 50 logarithmically increasing steps between 0.1 and 30 Hz at electrode Fz (upper) and Cz (lower). No statistical analysis was performed and interpretation of the plots is purely descriptive. Based on visual inspection the difference between Exp+ and Exp- was accompanied by a difference in theta power (3-7 Hz) in the first 500 ms. During the delay period, 500 ms after the predictive cue until feedback onset, a difference delta power (1-2 Hz is observed).
